# Vitamin D ointment for prevention of radiation dermatitis in breast cancer patients

**DOI:** 10.1038/s41523-017-0006-x

**Published:** 2017-03-31

**Authors:** Nicola J. Nasser, Shlomit Fenig, Amiram Ravid, Ariella Nouriel, Naama Ozery, Sara Gardyn, Ruth Koren, Eyal Fenig

**Affiliations:** 1 Institute of Oncology, Davidoff Center, Rabin Medical Center, Beilinson Hospital, Petah Tikva, Israel; 2 Department of Oncology, Wolfson Hospital, Holon, Israel; 3 The Basil and Gerald Felsenstein Medical Research Center, Sackler Faculty of Medicine, Tel Aviv University, Beilinson Campus, Rabin Medical Center, Petah-Tikva, Israel; 4 Sackler Faculty of Medicine, Tel Aviv University, Tel Aviv, Israel

## Abstract

Radiation dermatitis occurs frequently during adjuvant radiation therapy for breast cancer. Prevention of radiation dermatitis by applying various creams and ointments has a limited success, and Aqua cream which has urea as one of its active ingredients is used in many institutions as a preventive treatment. The primary goal of this study is to assess the effect of vitamin D (calcipotriol) ointment in prevention of radiodermatitis in breast cancer patients compared to Aqua cream. Twenty-three women with localized breast cancer who underwent breast-conserving surgery and opted to receive adjuvant radiotherapy to breast only were enrolled in this study. A cream containing an active vitamin D analog, calcipotriol (Daivonex), was randomly applied either to the medial or to the lateral half of the irradiated breast, while Aqua cream was applied to the complimentary half of the same breast along the whole treatment days, each day, after the delivery of radiation. Skin reaction was recorded and compared between the two halves of the breast. Vitamin D was well tolerated by patients with no local or systemic allergic reactions. Radiation dermatitis was not significantly different between both treatment arms. Topical vitamin D ointment is not superior to Aqua cream for prevention of radiation-induced dermatitis in women treated with adjuvant radiation for breast cancer.

## Radiation: Vitamin D not great for skin protection

Vitamin D ointment is no better than urea cream at preventing radiation-induced skin damage in breast cancer patients. Researchers in Israel led by Eyal Fenig from the Rabin Medical Center in Petah Tikva studied 23 women with localized breast cancer who underwent breast-conserving surgery and received adjuvant radiation to destroy any tumor cells left behind. Each day after their radiation therapy, the women applied an active vitamin D analog called calcipotriol (Daivonex) to half of their irradiated breast and a skin-hydrating, urea-containing ointment called Aqua cream to the other half of the same breast. The topical vitamin D ointment was well tolerated by the study participants. However, there was no noticeable difference in the effect of calcipotriol or Aqua cream for the vast majority of the women.

## Introduction

Breast cancer is the most frequent malignancy in women in the western world.^1^ Breast conserving surgery, followed by breast radiation was shown to be equivalent to total mastectomy, and turned to be a standard of care for patients with breast cancer.^2^ Adjuvant breast radiation decreases local recurrence of breast cancer, and is well tolerated. Acute side effects of breast radiation include radiation dermatitis which can range from mild to severe reactions.^3^ Prevention of radiation dermatitis using local therapies is so far disappointing.^4^

We have previously showed that in laboratory conditions, the active vitamin D metabolite, calcitriol, protects proliferating keratinocytes from the damage inflicted by ionizing radiation.^5^ Calcitriol inhibits both caspase-dependent and caspase-independent programmed cell death and increases the colony formation capacity of irradiated keratinocytes.^5^ These positive preliminary results, prompted us to evaluate the role of topical active vitamin D, calcipotriol, as a measure for preventing skin-induced radiation dermatitis in breast cancer patients.

## Results

### Patients clinical characteristics

**Fig. 1:**
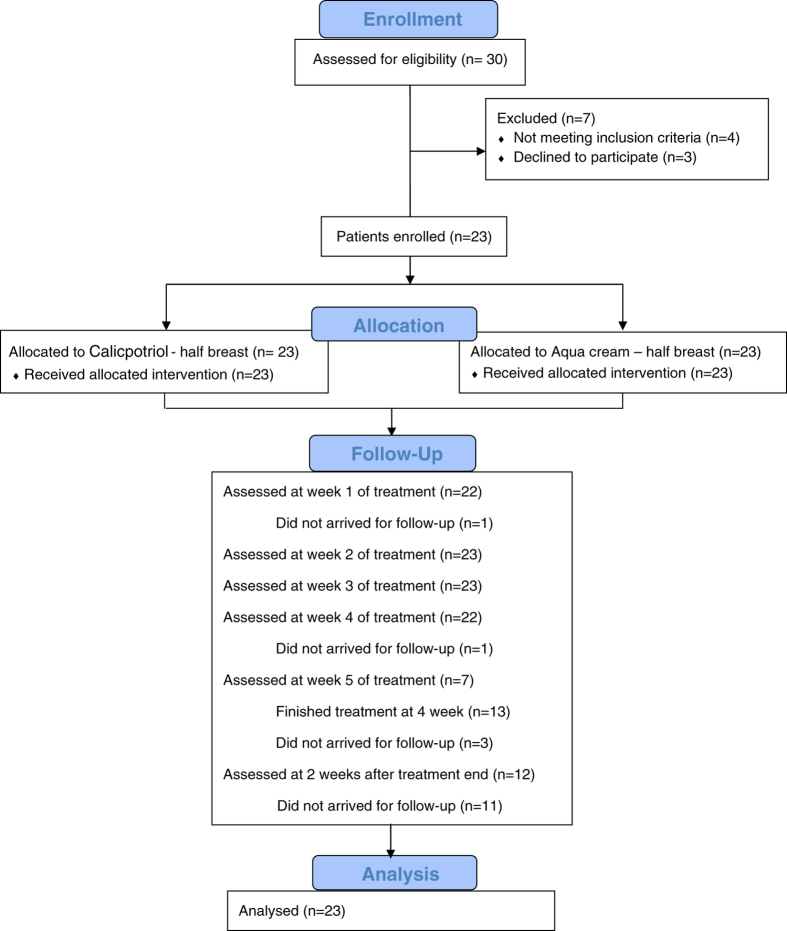
Consolidated Standards of Reporting Trials (CONSORT) flowchart

Thirty patients were assessed for eligibility. Four patients were excluded because they did not meet the eligibility criteria, and three patients declined to participate. From the 30 patients assessed, 23 patients were enrolled in the study and final analysis (Fig. 1). The trial was stopped after completion of the recruitment of the necessary number of patients. Mean age was 63 years (range, 37–74 years) (Table 1). Fifteen patients (65%) were of Ashkenazi, and eight patients (35%) were of Sephardic Jewish descent (Table 2). Seventeen of the 23 patients had no drug or food allergies. Four patients were allergic to penicillin, one to sesame, and one to sulfa. Six patients suffered from Ductal Carcinoma In Situ and 17 patients from Invasive Ductal Carcinoma. Clinical staging of all patients with invasive cancer was T1-2N0M0 (primary tumor diameter less than 5 cm). Twelve patients (52%) had radiation treatment to the left breast, while eleven patients (48%) had radiation treatment to the right breast.

**Table 1:** Patient’s characteristics

Characteristics	Daivonex (*n* = 23)
*Age (years)*	
Mean (± SD)	63 ± 8
Range	37–74
*Origin*	
Ashkenazi	15 (65%)
Sephardic	8 (35%)
*Breast (cup)*	
A	0 (0%)
B	5 (22%)
C	13 (57%)
D	4 (17%)
DD	1 (4%)
*Breast size*	
Small	0 (0%)
Medium	18 (78%)
Large	5 (22%)
*Extensive sun exposure*	
Yes	10 (43%)
No	13 (57%)
*Eye color*	
Green	2 (9%)
Blue-green	2 (9%)
Hazel	4 (17%)
Brown	14 (61%)
Black	1 (4%)
*Number of treatments*	
16	12 (52%)
25	9 (39%)
30	2 (9%)

**Table 2:** Breast skin toxicity according to RTOG scale in the parts of the breast treated with Daivonex or Aqua cream

Toxicity grade	Clinical Reaction	Daivonex (*n* = 23)	Aqua Cream (*n* = 23)
0	No reaction	0 (0%)	0 (0%)
1	Light erythema dry peeling and a decrease in sweat production	6 (26%)	5 (22%)
2	Scattered macular or papular eruption or erythema with pruritus or other associated symptoms	16 (70%)	17 (74%)
3	Extensive moist peeling and a pitting edema	1 (4%)	1 (4%)
4	Ulcers, bleeding, and necrosis	0 (0%)	0 (0%)

### Randomization of calcipotriol and Aqua cream to the treated parts of the irradiated breast skin

Fourteen patients (61%) applied calcipotriol to the lateral side of the breast, while nine patients (39%) applied calcipotriol to the medial side of the breast.

### Skin reaction

**Fig. 2:**
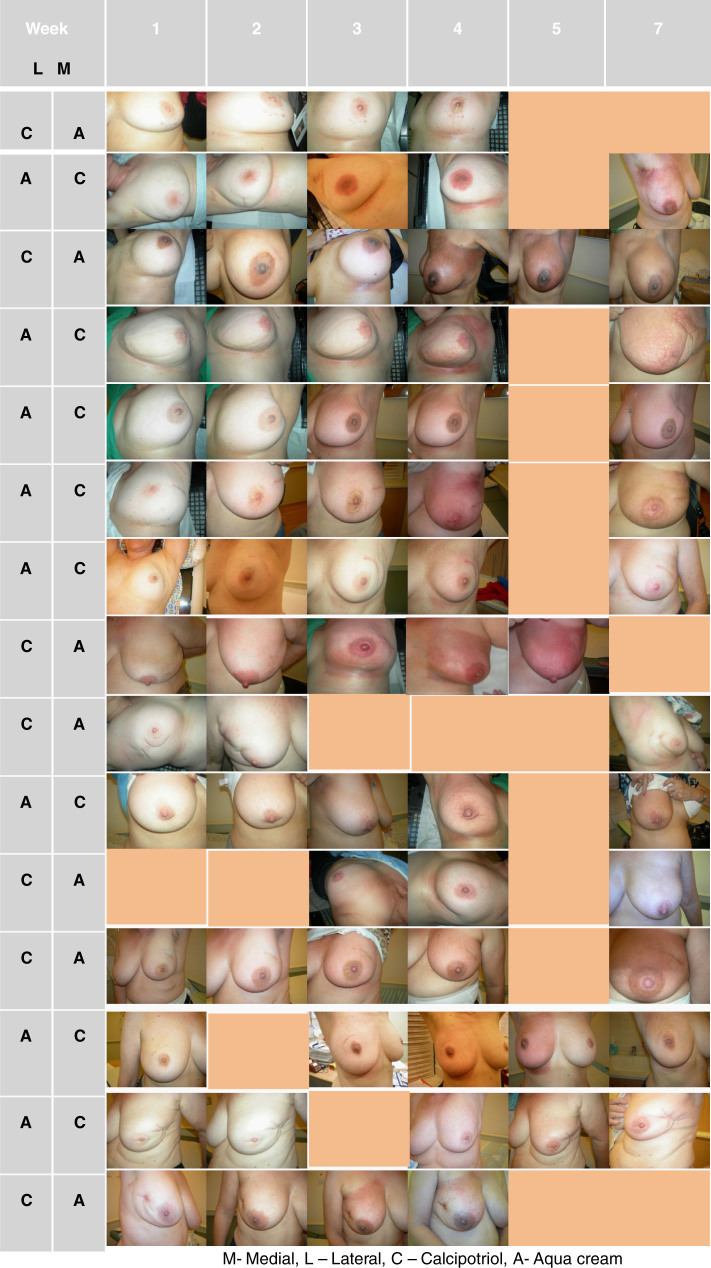
Breast skin reaction during radiation therapy. The irradiated breast was photographed each week, and at each follow-up visit. Each raw refers to the same patient. Lateral (L) or medial (M) parts of the irradiated breast were treated with Calcipotriol (C) or Aqua cream (A)

Vitamin D was well tolerated by patients with no localized or systemic allergic reactions. Skin toxicity was evaluated according to the Radiation Therapy Oncology Group (RTOG) scale for skin toxicity^6^ (Table 2). Photographic documentation of the breast was available for 15 patients (Fig. 2). The intensity of radiation dermatitis increased during treatment, and reached its highest intensity by the conclusion of radiation therapy. By the last day of radiation therapy, no grade 4 toxicity was detected. One patient suffered from grade 3 toxicity in both the calcipotriol treated breast half and the Aqua cream treated control half. Grade 2 skin toxicity was noted in 16 patients in both the part of the breast treated with calcipotriol and in the control part of the breast treated with Aqua cream. One patient suffered from grade 2 skin toxicity in the Aqua cream treated breast half, while having grade 1 skin reaction, in the breast half treated with the investigation cream, calcipotriol (Table 2).

Assessment of skin toxicity in the part of the breast treated with calcipotriol, compared to the part of breast treated with Aqua cream, according to the research physician and patient subjective judgment are described in Table 3. Clinical evaluation revealed that in 20 out of 23 patients (87%) there was no difference in the skin reaction between the calcipotriol and the Aqua cream treated skin parts of the breast. In two patients the skin reaction in the calcipotriol treated part of the breast was more intense compared to the Aqua cream treated part, while one patient had lesser skin toxicity in the calcipotriol treated breast part, compared to the breast part treated with Aqua cream (Table 3). There was no correlation between breast size, eye color, ethnic background or age, and radiation dermatitis.

**Table 3:** The patient and the treating physician compared the effect of Daivonex vs. Aqua cream on radiation dermatitis at the conclusion of the treatment

Measures of Assessment	Daivonex (*n* = 23)
*Patient’s impression*	
Detrimental effect	6 (26%)
No change	12 (52%)
Improved effect	5 (22%)
*Physician assessment*	
Detrimental effect	2 (9%)
No change	20 (87%)
Improved effect	1 (4%)

## Discussion

Radiation dermatitis is one of the most bothersome side effects of adjuvant radiation therapy for breast cancer.^6^ Here we show that topical vitamin D is not superior to the skin hydrating ointment, Aqua cream, in preventing radiation-induced skin toxicity. Vitamin D ointment was well tolerated, and breast skin areas treated with it suffered from similar skin toxicities compared to those inspected in the Aqua cream treated parts. The current study was powered to detect 20% improvement in skin toxicity, and was not powered to detect non-inferiority of vitamin D ointment. Due to the lower costs of skin hydrating ointments compared to the vitamin D ointments, our results show that there is no added benefit of using vitamin D ointment for the indication of preventing radiation dermatitis. Possible explanation to the discrepancy between our laboratory results^5^ and the current clinical data, is that in the laboratory study, vitamin D effect was tested on keratinocytes cell culture, while in the current study the skin tissue contain several cell types^7^ and layers on which, in the sum, vitamin D seems to have no radiation protecting effect. Another explanation is that the decreased cell death that potentially results from vitamin D may not be in correlation with radiation dermatitis.

Zhang et al. published a meta-analysis of 20 reports of clinical trials using topical agents for prevention and treatment of radiodermatitis.^8^ The analysis which included 3098 patients showed that the current topical agents do not prevent or treat radiation dermatitis effectively.^8^ Chan et al.^9^ provided an overview of six systematic reviews of published interventions for the prevention and management of radiation dermatitis.^10–15^ The authors found that the methodological quality of the studies varied, and methodological shortfalls in these reviews might create biases to the overall results or recommendations for clinical practice.^9^ Using experimental radiation in mice, Chen et al. demonstrated that topical steroid cream significantly attenuated radiation-induced inflammation.^16^ A double-blind randomized study showed that mometasone furoate, a potent corticosteroid cream, significantly reduces acute radiation dermatitis.^17^ Several other studies showed benefit for mometasone furoate.^18, 19^ Schmuth et al*.* ^20^ randomized patients treated with radiotherapy to prophylactic methylprednisolone vs. dexpanthenol but didn’t proved added value of corticosteroids. A phase III randomized trial comparing *Calendula officinalis* with trolamine for the prevention of acute dermatitis during radiation therapy for breast cancer showed an improved outcomes with *Calendula*,^21^ but results from another randomized blinded trial showed no differences between *Calendula* cream and aqueous cream in the prevention of acute radiation skin reactions.^22^ Recently, several studies focused on changing the radiation technique for adjuvant breast cancer in an effort to reduce skin toxicity.^23, 24^ A multicenter randomized trial showed that Intensity Modulated Radiation Therapy (IMRT) for the breast cancer results in less radiation dermatitis.^24, 25^ Several systemic approaches for preventing radiation induced dermatitis were described, focusing on antioxidant nutrients,^26, 27^ but currently there is no randomized study which proved benefit of such approach.

In conclusion, topical vitamin D is not superior to the skin hydrating ointment, Aqua cream, in the prevention of radiation induced skin toxicity during adjuvant radiation therapy after breast conserving surgery.

## Materials and methods

### Ethics statement

The Ethics Committee of Rabin Medical Center approved this study. Written informed consent was obtained from each participant prior to study entry. Consent to publish unidentifiable photos of the irradiated breast was obtained from each patient. ClinicalTrials.gov Identifier: NCT00445250.

### Eligibility

The study was performed at the Rabin Medical Center (Petach Tikva, Israel) between April 2007 and May 2012. Inclusion criteria for the study were women aged 18 to 75 years with a confirmed histological diagnosis of localized breast cancer. All patients were after breast lumpectomy, and scheduled to receive adjuvant radiotherapy. Patients were excluded from the study if they had scleroderma, large breast with an inter-field of more than 25 cm, or prior radiotherapy to the same breast. Patients with indication to lymph node irradiation were not included in this study. Patients were enrolled by the treating radiation oncologist.

### Radiation therapy

Treatment simulation was performed using computerized tomography planning. Radiation therapy to the involved breast was delivered by two tangential fields. Radiation therapy was delivered as a single fraction per day, 5 days a week. Radiation dose was either 42.72 Gy in 16 fractions or 50 Gy in 25 fractions. When indicated, a boost was provided to the tumor bed, to a total dose of 10 Gy administered in 5 fractions of 2 Gy each.

### Topical ointments

The medial and lateral parts of the irradiated breast were randomly assigned by coin flipping, by the study coordinator, to receive either Daivonex (LEO Pharmaceutical Products Ltd., Ballerup, Denmark) or Aqua cream. After 15 min of receiving the daily radiation dose, Daivonex was applied to half of the breast and Aqua cream to the other half, by the patient under supervision of the study nurse. The study arms were open to the patients and the investigators.

### Clinical evaluation

Adverse skin effects were assessed by a physician and a nurse according to the RTOG scoring system.^6^ A questionnaire about the efficacy and safety of the cream was given to each patient. Evaluation was performed at 1, 2, 3, 4, 5, and 7 weeks following the initiation of treatment. The irradiated breast was photographed at each follow-up visit.

### Statistical analysis

Sample size was calculated to detect a change of 20% or more in radiation dermatitis, and it was calculated that 20 assessable patients were required. An interim analysis was planned to stop the trial in case any detrimental effect, or grade 4 or 5 toxicities, were observed among the first eight treated patients. Statistical analysis was performed using the statistical soft ware program Microsoft Office Excel 2007.
